# Supplementary material for: High-resolution gridded estimates of population sociodemographics from the 2020 census in California
Source: PLoS One. 2022 Jul 14;17(7):e0270746. doi: 10.1371/journal.pone.0270746 (PMC9282657; doi:10.1371/journal.pone.0270746)
Supplement: S1 Table — (DOCX) [file pone.0270746.s001.docx]

**S1 Table**. **Land use codes from tax parcel dataset identified as residential**

| **Parcel Land Use Code** | **Parcel Count** | **Total Area [km2]** | **% Res. Area** |
| --- | --- | --- | --- |
| APARTMENT HOUSE (100+ UNITS) | 1724 | 16.17 | 0.05% |
| APARTMENT HOUSE (5+ UNITS) | 112705 | 491.82 | 1.59% |
| APARTMENTS (GENERIC) | 32154 | 92.30 | 0.30% |
| CLUSTER HOME (RESIDENTIAL) | 51547 | 37.06 | 0.12% |
| COMM/OFC/RES MIXED USE | 18307 | 57.81 | 0.19% |
| CONDOMINIUM (RESIDENTIAL) | 330047 | 228.86 | 0.74% |
| COOPERATIVE (RESIDENTIAL) | 184 | 0.87 | 0.00% |
| DORMITORY, GROUP QUARTERS (RESIDENTIAL) | 86 | 3.57 | 0.01% |
| DUPLEX (2 UNITS, ANY COMBINATION) | 236620 | 436.01 | 1.41% |
| FRATERNITY HOUSE, SORORITY HOUSE | 263 | 3.10 | 0.01% |
| GARDEN APT, COURT APT (5+ UNITS) | 275 | 0.83 | 0.00% |
| HIGHRISE APARTMENTS | 20 | 0.08 | 0.00% |
| HOMES (RETIRED; HANDICAP, REST; CONVALESCENT; NURSING) | 3959 | 34.73 | 0.11% |
| MANUFACTURED, MODULAR,  PRE-FABRICATED HOMES | 9252 | 96.33 | 0.31% |
| MISC RESIDENTIAL IMPROVEMENT | 16837 | 689.14 | 2.22% |
| MOBILE HOME | 140782 | 2185.87 | 7.06% |
| MOBILE HOME PARK, TRAILER PARK | 5251 | 220.52 | 0.71% |
| MULTI-FAMILY DWELLINGS  (GENERIC, ANY COMBINATION 2+) | 72299 | 280.49 | 0.91% |
| PLANNED UNIT DEVELOPMENT (PUD) (RESIDENTIAL) | 143406 | 130.58 | 0.42% |
| QUADRUPLEX (4 UNITS, ANY COMBINATION) | 63375 | 66.32 | 0.21% |
| RESIDENTIAL (GENERAL) (SINGLE) | 25325 | 717.73 | 2.32% |
| RESIDENTIAL COMMON AREA  (CONDO/PUD/ETC.) | 18792 | 118.42 | 0.38% |
| RESIDENTIAL INCOME (GENERAL)  (MULTI-FAMILY) | 18794 | 123.72 | 0.40% |
| RURAL RESIDENCE (AGRICULTURAL) | 166093 | 12004.65 | 38.75% |
| SINGLE FAMILY RESIDENTIAL | 7255233 | 12849.40 | 41.48% |
| STORES & APARTMENTS | 1599 | 1.80 | 0.01% |
| TIMESHARE (RESIDENTIAL) | 1218 | 2.23 | 0.01% |
| TOWNHOUSE (RESIDENTIAL) | 44892 | 9.32 | 0.03% |
| TRIPLEX (3 UNITS, ANY COMBINATION) | 60875 | 73.61 | 0.24% |
