# Supplementary material for: High-resolution gridded estimates of population sociodemographics from the 2020 census in California
Source: PLoS One. 2022 Jul 14;17(7):e0270746. doi: 10.1371/journal.pone.0270746 (PMC9282657; doi:10.1371/journal.pone.0270746)

**S2 Figure.** **False negatives in the Microsoft building footprint data.**

Examples shown in urban and rural contexts. Locations were chosen based on the presence of false negatives and do not generally reflect the typical proportion of false negative instances around the state. (Satellite base imagery source: USGS (NAIP) from The National Map)


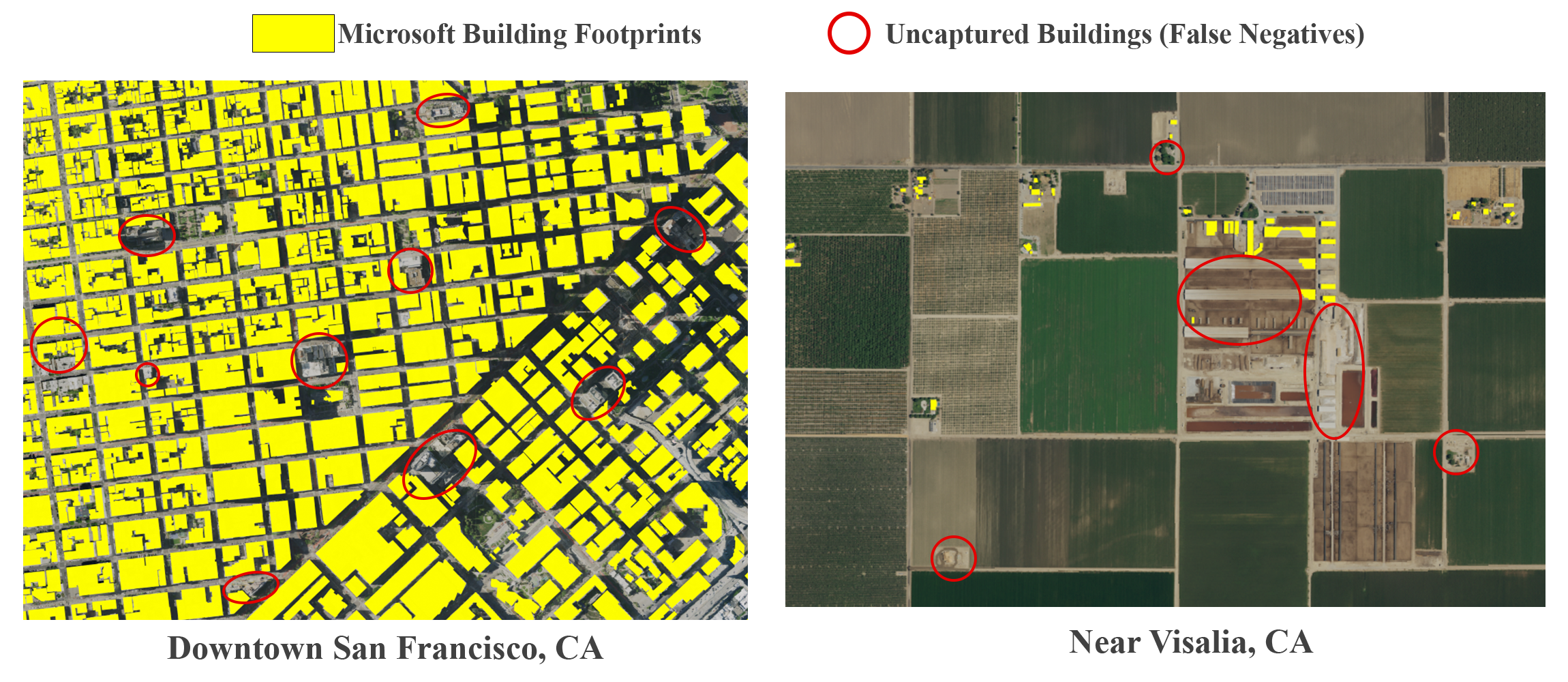

Supplement: S1 Fig — Examples shown in urban and rural contexts. Locations were chosen based on the presence of false negatives and do not generally reflect the typical proportion of false negative instances around the state. (DOCX) [file pone.0270746.s002.docx]
