# Supplementary material for: High-resolution gridded estimates of population sociodemographics from the 2020 census in California
Source: PLoS One. 2022 Jul 14;17(7):e0270746. doi: 10.1371/journal.pone.0270746 (PMC9282657; doi:10.1371/journal.pone.0270746)

**S3 Figure**. **Examples of 1-acre residential parcels.**

1-acre (~4050 km^2^) was used as the upper area threshold for low-density residential parcels. (Satellite base imagery source: USGS (NAIP) from The National Map)


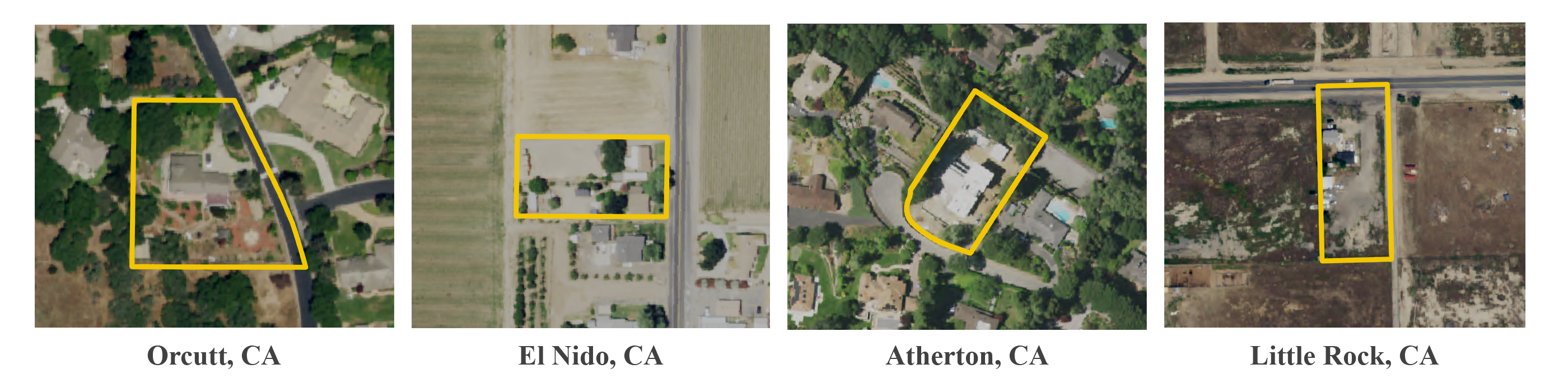

Supplement: S2 Fig — 1-acre (~4050 km2) was used as the upper area threshold for low-density residential parcels. (DOCX) [file pone.0270746.s003.docx]
