# Supplementary material for: High-resolution gridded estimates of population sociodemographics from the 2020 census in California
Source: PLoS One. 2022 Jul 14;17(7):e0270746. doi: 10.1371/journal.pone.0270746 (PMC9282657; doi:10.1371/journal.pone.0270746)

**S4 Figure**. **Percent errors of block group-constrained CA-POP population grid relative to census block population observations.**

Values represent the percent difference between block-level estimates from the block group-constrained CA-POP total population grid and census block population values. Note that these errors do not reflect the block-level errors of the final CA-POP grids themselves, as those were constrained by the block-level census observations, which by definition makes block-level errors zero. Errors in the final grids instead occur at the sub-block level of population apportionment, for which there are no ground-truth population observations available for assessing CA-POP’s ability to apportion population within blocks. (Ocean boundary layer source: Natural Earth).


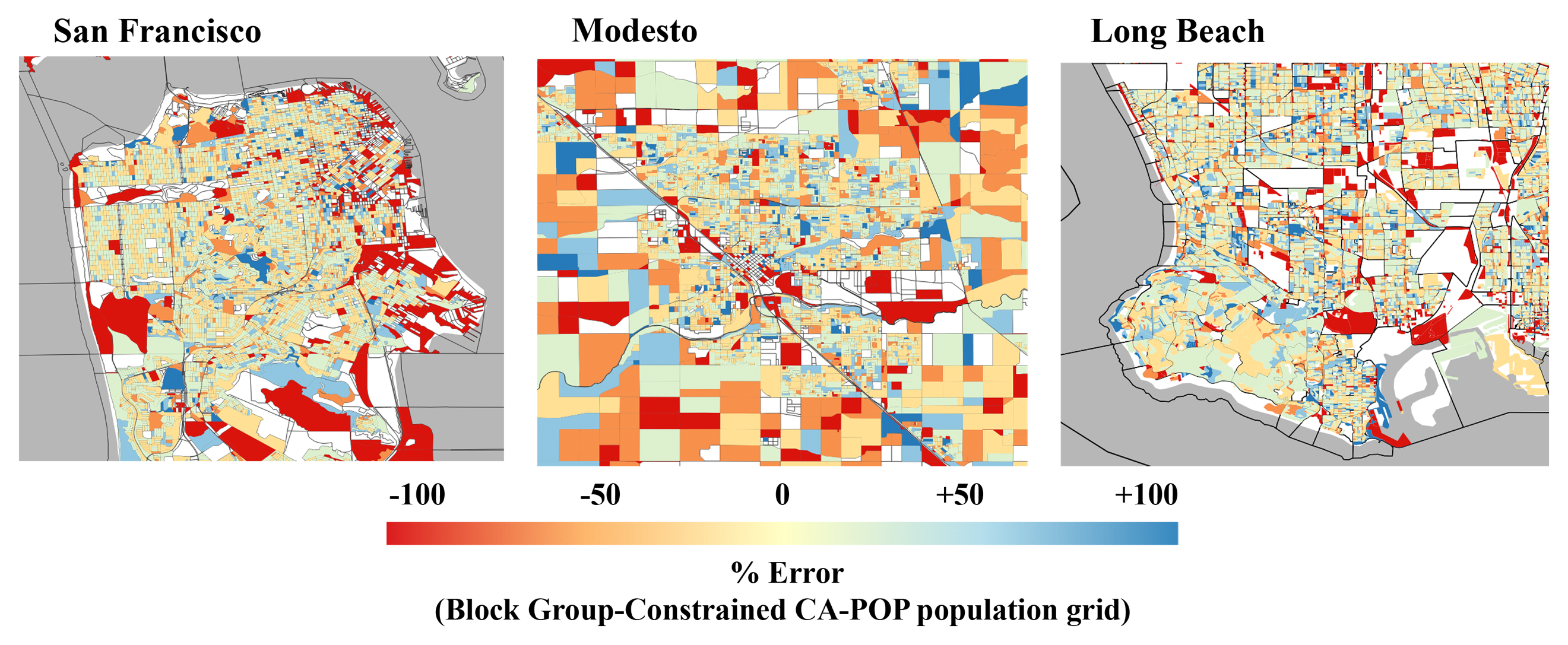

Supplement: S3 Fig — Values represent the percent difference between block-level estimates from the block group-constrained CA-POP total population grid and census block population values. Note that these errors do not reflect the block-level errors of the final CA-POP grids themselves, as those were constrained by the block-level census observations, which by definition makes block-level errors zero. Errors in the final grids instead occur at the sub-block level of population apportionment, for which there are no ground-truth population observations available for assessing CA-POP’s ability to apportion population within blocks. (DOCX) [file pone.0270746.s004.docx]
